# Identifying Patient-Reported Outcome Measures (PROMs) for Routine Surveillance of Physical and Emotional Symptoms in Head and Neck Cancer Populations: A Systematic Review

**DOI:** 10.3390/jcm10184162

**Published:** 2021-09-15

**Authors:** Sheilla de Oliveira Faria, Gillian Hurwitz, Jaemin Kim, Jacqueline Liberty, Kimberly Orchard, Geoffrey Liu, Lisa Barbera, Doris Howell

**Affiliations:** 1 Faculdade de Medicina FMUSP, Universidade de Sao Paulo, Sao Paulo 01246-903, Brazil; 2 Department of Supportive Care, Princess Margaret Cancer Centre, Toronto, ON M5G 2M9, Canada; Doris.Howell@uhn.ca; 3 Lawrence S. Bloomberg Faculty of Nursing, University of Toronto, Toronto, ON M5T 1P8, Canada; 4 Cancer Care Ontario, Toronto, ON M5G 2L7, Canada; gillian.hurwitz@cancercare.on.ca (G.H.); Jaemin.Kim@ontariohealth.ca (J.K.); jacqueline.liberty@cancercare.on.ca (J.L.); orchard.kimberly@gmail.com (K.O.); 5 Department of Medical Oncology and Hematology, Princess Margaret Cancer Centre, Toronto, ON M5G 2M9, Canada; Geoffrey.Liu@uhn.ca; 6 Dalla Lana School of Public Health, Toronto, ON M5T 3M7, Canada; 7 Temerty Faculty of Medicine, Toronto, ON M5S 1A8, Canada; 8 Department of Medical Biophysics, University of Toronto, Toronto, ON M5S 1A1, Canada; 9 Tom Baker Cancer Centre, University of Calgary, Calgary, AB T2N 1N4, Canada; Lisa.Barbera@albertahealthservices.ca

**Keywords:** head and neck cancer, patient-reported outcome, patient-reported measures, symptoms, side effects, adult, cancer survivors

## Abstract

The aims of this review were to identify symptoms experienced by head and neck cancer (HNC) patients and their prevalence, as well as to compare symptom coverage identified in HNC specific patient-reported outcome measures (PROMs). Searches of Ovid Medline, Embase, PsychInfo, and CINAHL were conducted to identify studies. The search revealed 4569 unique articles and identified 115 eligible studies. The prevalence of reported symptoms was highly variable among included studies. Variability in sample size, timing of the assessments, and the use of different measures was noted across studies. Content mapping of commonly used PROMs showed variability and poor capture of prevalent symptoms, even though validation studies confirmed satisfactory reliability and validity. This suggests limitations of some of the tools in providing an accurate and comprehensive picture of the patient’s symptoms and problems.

## 1. Introduction

In 2020, GLOBOCAN estimated 932,000 new cases of head and neck cancer (HNC) and 467,000 deaths in 2020 worldwide [1]. HNC refers to a group of cancers including oral cancer, pharynx, larynx, paranasal sinuses and nasal cavity, and salivary glands [2,3]. Due to the location of cancer and type of treatment, HNC patients experience unique oral morbidity and related symptoms such as dysphagia, xerostomia, trismus, osteoradionecrosis, mucositis, lymphedema, and sialadenitis [4,5]. They may also experience changes in appearance and speech, decreased neck mobility, and shoulder dysfunction [6,7]. These changes can affect self-esteem and body image, sexuality, social anxiety, physical functioning, and quality of life (QOL), leading to high levels of psychological distress [4,8].

Patient-reported outcome measures (PROMs) are used in healthcare systems to determine the impact of disease and treatment on the patient and to estimate disease burden across a population [9]. PROMs are standardized, validated questionnaires completed by patients to measure their symptoms, perceptions of health status, and/or functional well-being [10]. PROMs should capture the most prevalent symptoms and treatment effects experienced by HNC patients. However, it is unclear to what extent PROMs map to specific problems of HNC patients and if they are psychometrically sound. Selection of a core set of condition-specific PROMs for routine capture specific to HNC and its treatment effects is critical for guiding patient management in routine care, for estimating disease burden, and for value-based performance measurement in the cancer system.

A preliminary review of the literature identified three previously conducted systematic reviews on PROMs for assessing QOL in HNC populations, but none have mapped PROMs to identify their capture of prevalent physical and emotional symptoms or other problems in this population [11,12,13]. Thus, the aims of this study were to (1) explore the prevalence of symptom burden and treatment effects in HNC, (2) identify relevant PRO domains and PROMs specific to HNC, and (3) evaluate psychometric properties to recommend use in routine care.

## 2. Materials and Methods

**Figure 1:**
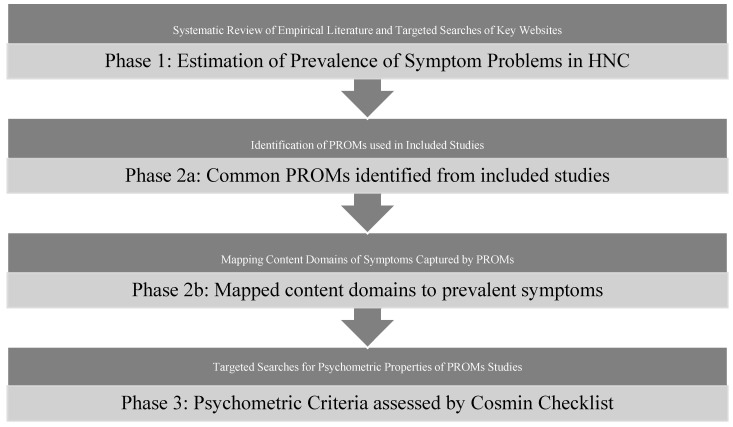
Phases to identify PROMs for routine surveillance of symptoms in HNC.

This systematic literature review focused on HNC patients undergoing treatment (surgery, radiation, and/or chemotherapy). There were three phases of work: (1) a systematic review of the literature to identify prevalence of symptom burden, (2) identification of common PROMs with mapping of domains and items to HNC specific symptoms and comparison of PROM-content across measures, and (3) review of psychometric properties of identified PROMs (Figure 1).

### 2.1. Search Strategy

Systematic searches of electronic databases were conducted in MEDLINE, EMBASE, PsychINFO, and CINAHL to identify studies that reported prevalence rates for HNC symptoms. Gray literature sources were also searched and included National Health Service in England (NHS), American Society of Clinical Oncology (ASCO), International Society for Pharmacoeconomics and Outcomes Research (ISPOR), Integrating the Healthcare Enterprise (IHE), and Cancer Australia websites. MEDLINE and PubMed searches were also conducted to obtain validation studies of the most commonly used PROMs, specifically the Head and Neck Radiotherapy Questionnaire (HNRT-Q), Quality of Life Questionnaire (QLQ)—Rathmell, Quality of Life (QOL)—Thyroid, Functional Assessment of Cancer Therapy—Nasopharyngeal (FACT-NP), Oral Mucositis Quality of Life Measure (OMQOL), Functional Status in Head and Neck Cancer—Self Report (FSH&N-SR), and MD-Anderson Symptom Inventory—Head and Neck (MDASI-H&N). Detailed search strategies are displayed in Appendix A.

### 2.2. Selection of Studies

All titles and abstracts were screened independently by one of two reviewers (K.O., S.O.F.), with a portion of the studies double-screened by a second reviewer (J.L.). Forward reference searching was performed on all studies and for any systematic reviews to identify additional primary studies. We followed the steps for screening of studies specified by Higgins and Deek [14]: (1) we merged all references into a reference management database and de-duplicated; (2) we examined titles/abstracts and excluded obviously irrelevant studies; (3) we reviewed full papers for eligibility, but did not contact authors of papers. For phase 1 and 2, studies were included if (1) the prevalence rates for symptoms were reported for HNC patients, and (2) there were a minimum of 10 cases. For phase 3, studies were included if they were PROMs validation studies for the common HNC measures identified for further review. The HNC measures chosen for phase 3 were those that were cited most frequently in prevalence studies and based on their coverage of physical and emotional symptoms specific to HNC.

We excluded editorials, commentaries, and conference abstracts, studies focused on generic health-related quality of life (HRQoL) measures and did not restrict by study type.

### 2.3. Data Extraction and Assessment

Data were extracted independently by three reviewers (K.O., or K.J., or S.O.F.) and assessed by a second reviewer (J.L.). Extraction was guided by a template developed for this review and approved by all authors that included data on study purpose, study design, population characteristics (sample size, disease sites, treatment types, stage, and age characteristics at diagnosis), characteristics of measurement tools used, and prevalence data.

### 2.4. Content Analysis of the PROMs

Content domains and items of the PROMs was extracted and synthesized as per the methods described by Macefield et al. [15]. First, verbatim names for the scales and single items, as termed by the PROM developers, were extracted and listed. Scales and items with identical or similar names were documented, grouped, counted, and compared for consistency. Each group of scales and items was identified by their conceptual domains and mapped onto the physical and emotional symptom domains, i.e., emotional symptoms or sub-domains (i.e., sadness or depression) or other problems identified from the phase 1 review of prevalence. Two members of the team verified the mapping of domains and items from PROMs to the symptom problems.

## 3. Results

**Figure 2:**
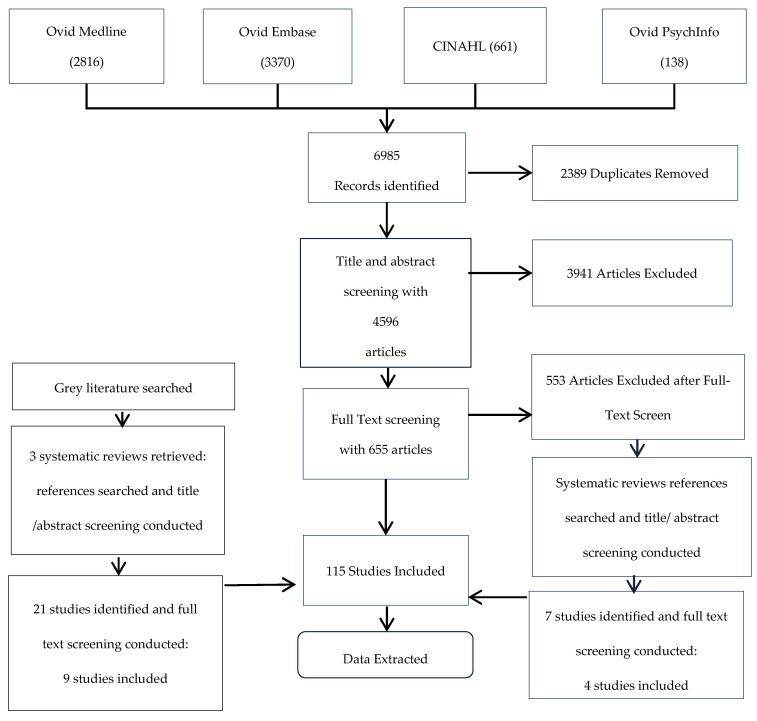
PRISMA flow diagram.

As shown in Figure 2 (PRISMA chart), our search strategy identified 4569 unique articles. Of these, 115 studies that examined the prevalence of symptoms in HNC were included.

### 3.1. Characteristics of the Included Studies

Studies were limited to 2004 onwards, with 88 (76%) of the included studies published after 2010, of which there were 63 cross-sectional studies, 45 prospective cohort studies, 4 retrospective cohort studies, 2 controlled studies, and 1 mixed-methods study. Studies either included patients across different cancer stages (I–IV) or did not specify the cancer stage. Study characteristics are provided in Supplementary Table S1.

### 3.2. Emotional Distress and Psychosocial Symptoms

As shown in Table 1, emotional distress and psychosocial symptoms were the most common issues identified in HNC, including depression (*n* = 22 studies), sadness (*n* = 5 studies), anxiety (*n* = 20 studies), worry (*n* = 3 studies), emotional distress (*n* = 7 studies), satisfaction with appearance (*n* = 4 studies), and avoidance of social interactions (*n* = 3 studies).

#### 3.2.1. Depression

Depression was commonly identified in many included studies, although study heterogeneity precluded meta-analysis. Sample sizes ranged from 23 to 1217 patients. Variability in rates of depression were noted and ranged from 2% to 84% due to differential timing of assessments, different scales, and different threshold values. For example, across different studies, depression was evaluated using any of the following instruments: Hospital Anxiety and Depression Scale (HADS) [16,17,18,19,20,21,22,23,24,25,35,36], the Beck Depression Inventory (BDI) [26,27,28], the short-form of the Geriatric Depression Scale (GDS-SF) [29], the Neuropsychiatric Inventory Questionnaire (NPI-Q) [30], the Research Diagnostic Criteria Schedule for Affective Disorders and Schizophrenia (RDC SADS) [31], the University of Washington Quality of Life Mood scale (UWQOL-mood) [32], and the Patient Health Questionaire-8 (PHQ-8) [4]. Chen et al. [16] evaluated the prevalence of depression over time using the HADS-D (cut-off score of ≥8) and the BDI (cut-off score ≥ 14) and reported a 13% difference in the number of patients with depression as identified by the HADS-D and the BDI at pre-treatment, a 5% difference in prevalence during treatment, and a 12% difference post-treatment between instruments. Further to this, Katz et al. [31] applied the research diagnostic criteria (RDC) clinical diagnostic criteria for depression to a sample of HNC patients and used these results to compare the sensitivity, specificity, and positive predictive values of different threshold scores for different instruments. As may be expected, each instrument and associated cut-off scores evaluated had varying levels of performance [31].

Levels of depression in HNC appear to be independent of age, sex, disease site, and cancer stage [26,31,33]. Karnell et al. [26] found that higher levels of pre-treatment depressive symptoms were the only factor in multivariate analysis that was associated with persistently high levels of post-treatment depressive symptoms (odds ratio of 1.762; *p* < 0.01). An increasing trend as treatment progressed in both the prevalence and severity of depression was noted across most studies; this trend generally reversed and declined post-treatment [16,27,28,47]. McDowell et al. [23] found depression to be prevalent in one-quarter of patients with nasopharyngeal carcinoma even after 4 years of being disease-free after definitive intensity-modulated radiation therapy (IMRT). Given that depression is closely linked to physical symptom severity, this pattern of increasing prevalence and severity as treatment progresses was not surprising [17,47].

#### 3.2.2. Sadness

Sadness was reported in five studies, ranging between 8% and 82% [37,38,39,40,41]. There was no consistency in terms of measurement tools used. One study reported that patients who underwent surgery were more likely to report being sad than those who had received chemotherapy (20% vs. 14% prevalence respectively) [38].

#### 3.2.3. Anxiety

Twenty studies reported on prevalence of anxiety in HNC [4,16,17,18,19,20,21,22,23,25,30,33,35,36,37,42,43,44,45,46]. Sample sizes ranged from 23 patients to 229 patients and prevalence rates ranged from 1% to 97.5%. Similar to findings for depression, differences in instruments used and thresholds may explain this variability. The majority of studies used HADS-A to evaluate anxiety [16,18,19,20,23,43,44,46], while other studies used the UW-QOL [42], Palliative Symptom Impact List (Pal-SI) [37], NPQ-I [57], Patient Concern Inventory (PCI) [45] and Generalized Anxiety Disorder Questionnaire 2 (GAD-2) [17]. In general, the pattern of anxiety remained stable throughout the trajectory of illness, with little difference reported from pre- to post-treatment. Almonacid et al. reported a pre-treatment prevalence of 70%, which increased to 97% one week after treatment and dropped to almost the same prevalence as pre-treatment two weeks after treatment ended (72%) [46]. Neilson et al. reported a pre-treatment prevalence of 20%, 17% three weeks after treatment, and 22% at 18 months [17]. Neither Almonacid et al. nor Neilson et al. reported whether these changes in prevalence over time were statistically significant. Likewise, Kelly et al. reported pre, during, and post-treatment prevalence, with little differences found (34%, 34.5%, 34%, respectively) [22]. The prevalence of anxiety in HNC populations is still significantly higher than in the general population [21].

#### 3.2.4. Emotional Distress

Seven studies examined emotional distress among HNC patients [38,40,41,48,49,50,51]. Measures and cut-off scores for identifying clinically significant distress varied among studies. The Distress Thermometer (DT) was used in three studies, with cut-off scores ranging from 3 to 5 [48,49,50]; two studies reported an overall prevalence of distress in 50% of the population surveyed [48,49], while Wells et al. [50] reported a prevalence of 35% for mild distress and 33% for moderate/severe distress. Three studies that used MDASI-HN reported an overall prevalence of distress ranging between 14% and 86% [38,40,41]. Although treatment type (surgery versus chemotherapy) was not found to be a predictor of distress [38], one study reported that disease site—cutaneous (involvement of the lips, eyelids, ear, nose or face) versus non-cutaneous (larynx, oral/nasal cavity, glands, oro/nasopharynx) was a significant predictor [48].

#### 3.2.5. Other Emotional Symptoms

Three studies reported on worry, with prevalence ranging between 30% and 62% [30,37,47]. Unlike most other symptoms, prevalence was highest before treatment (62%), and dropped significantly, as treatment progressed (38% at 5 weeks during treatment, and 33% at 12 weeks after treatment) [47,58]. Others, such as Bond et al., reported prevalence of emotions of apathy and indifference in 56.5% of patients and agitation and aggression in 52.5% [30].

#### 3.2.6. Satisfaction with Appearance

Four studies of HNC patients undergoing surgery examined patient satisfaction with appearance [45,52,53,54]. In two studies, approximately 75% of patients reported either some type of body image concern or dissatisfaction with appearance [52,53]. There was also a significant difference in pre-surgical levels of satisfaction compared to post-surgical levels, with patients reporting significantly lower levels of satisfaction post-surgery [52,54]. One study used PCI and reported an overall prevalence of 89% [45].

#### 3.2.7. Avoidance of Social Interactions

Three studies provided estimates for the prevalence of social dysfunction [53,55,56]. In one study, 38% of patients reported avoidance of social activities due to appearance, speech or eating concerns [53]. Dwivedi et al. found that 41% of oral cancer patients and 16% of oropharyngeal cancer patients reported avoiding social activities due to speech alone [55].

#### 3.2.8. Substance Abuse Problems

Duffy et al. examined problem drinking and smoking: 16% of patients screened positive for problem drinking, while 30% had smoked cigarettes within the last month [29]. The study found that smokers and problem drinkers were more likely to be younger, not married, and within one year of diagnosis. The authors also reported that while smoking was negatively associated with all quality of life scale domains, problem drinking was not associated with any.

#### 3.2.9. Delirium

Bond et al. examined the prevalence of delirium among HNC patients undergoing chemotherapy [59]. Among 58 patients who completed their 3-month follow-up, 18 (31%) self-reported experiencing delirium at some point during their chemotherapy, while only 9% of patients were diagnosed with delirium using the Confusion Assessment Method (CAM). No patients reported experiencing delirium before or after treatments.

### 3.3. Physical Symptoms

Several studies evaluated prevalence of physical symptoms in HNC (Table 2).

#### 3.3.1. Eating and Nutritional Status

##### Dysphagia

A total of 35 studies assessed dysphagia, or difficulty swallowing [4,5,37,38,39,41,42,45,56,58,60,61,62,63,64,65,66,67,68,69,70,71,72,73,74,75,76,77,78,79,80,81,82,83,84]. Prevalence of dysphagia ranged between 0% and 100% across studies. Sample size range was between 12 and 8002. The University of Washington Quality of Life (UW-QOL) questionnaire swallowing subscale [42,63,65,71], the M.D. Anderson Dysphagia Inventory (MDADI) [41,62,68,125], and the Common Terminology Criteria for Adverse Event [74,77,78,79,80,81] were the most commonly used instruments to assess dysphagia. Jager-Wittenaar et al. reported that approximately 28% of patients (oral, pharynx, and larynx) experienced dysphagia at diagnosis, likely as a result of the disease itself [60]. Studies that compared the symptoms before, during, and after radiotherapy found HNC reported greater problems with swallowing as treatment progressed [58,61,67,68]. Symptoms also persisted well beyond treatment and did not return to baseline levels until 6 or more months post-radiotherapy [56,61,67]. Similar findings have also been reported pre- versus post-surgical resection [42,66]. Longer-term follow-up studies have suggested that the prevalence of dysphagia remains higher for patients who have undergone radiotherapy (15–95% prevalence at 12 months follow-up) [58,61,67] or multimodal treatments with chemotherapy and radiotherapy (75–79% prevalence at 6–60 months follow-up) [62,76] compared to those who underwent surgery alone (51% prevalence 28 months follow-up) [56]. Receiving multiple treatment modalities was identified as an important predictor of dysphagia [38,75]. Patients who received concomitant chemotherapy and radiotherapy generally experience a higher prevalence of dysphagia compared to those who undergo radiotherapy [61] or surgery alone [64]. However, radiotherapy alone is also a significant predictor of dysphagia [63,66]. Even type of radiotherapy was found as a predictor of dysphagia [76,77]. The absorbed dose to specific regions also appears relevant in the development of acute RT-related dysphagia [72]. Disease site may play an important factor in swallowing function. In a cross-sectional population-based study, Francis et al. found that the prevalence of dysphagia varied by disease site [64]. Compared to oral cancer, patients with cancer of the oropharynx, hypopharynx, or larynx were significantly more likely to have dysphagia [64]. In contrast, Rinkel et al. found that patients treated for a laryngeal or hypopharyngeal tumor had significantly better scores compared to patients treated for an oral cavity, oropharyngeal tumor, or nasopharynx tumor on the total [76]. More generally, Suarez-Cunquiero et al. found that patients with tumors located in the floor of the mouth and oropharynx experienced greater burden than other disease sites. In the same study, earlier stage disease was also found to be associated with better swallowing scores [66]. Difficulty swallowing also had a negative effect on quality of life [34,56,61] and weight loss [60,75]. Sixty-two percent of patients avoided eating with others, and 37% felt embarrassed at meal times due to their dysphagia [56]. In patients >65 years old during initial treatment, the development of severe late dysphagia was significantly more frequent [83].

##### Xerostomia/Saliva Function

Prevalence of xerostomia was reported in 23 studies [5,38,39,40,41,42,45,58,65,67,69,71,73,77,78,79,80,85,86,87,88,89,90]. Pre-treatment prevalence of xerostomia was relatively low among HNC patients (4%-18%), indicating it that was likely treatment-induced [38,40,42,85]. Haisfield-Wolfe et al. found a sharp increase in the prevalence of xerostomia during the course of radiotherapy (71% at week 1, 91% at week 5, and 95% at week 9) [58]. Post-treatment prevalence of xerostomia remained high, with some reduction in prevalence rates noted (rate range 64 to 44%) at one year [65,83].

As may be expected, treatment type was a significant predictor of xerostomia. Arribas et al. reported that after induction chemotherapy (iCT), the prevalence was 15%, and 45% after RT [73]. Gunn et al. reported that the patients scheduled to undergo radiotherapy who had completed prior chemotherapy or surgery experienced higher prevalence of xerostomia (11.9% and 14% respectively) than untreated patients (5.5%) [38]. Radiotherapy alone was a significant predictor of xerostomia [86].

##### Trismus

Fourteen studies examined the prevalence of trismus [24,45,69,77,86,90,91,92,93,94,95,96,97,98]. Assessment tools for trismus varied across studies but the most commonly used was the Maximal Interincisal Distance/Opening (MIO/MID) with a cut-off value ≤ 35 mm [24,91,92,93,94,95,96,97,98]. The remaining studies assessed trismus using the EORTC QLQ C-30 trismus subscale [69], the Mandibular Function Impairment Questionnaire (MFIQ) [86], and the PCI [45]. Prevalence rates were variable pre-treatment (3–41%) and after treatment (12–57%) [91,92,93,94,98]. Lee et al. examined prevalence pre- and post-surgery and found that rates continued to increase over time (41%, 71%, and 79% at pre-op, 6 weeks post-surgery, and 6 months post-surgery, respectively) [91]. A similar pattern was reported by Lindblom et al. comparing before and after radiotherapy (3%, 38%, and 41% at pre-radiotherapy, post-radiotherapy, and at a median of 66 months, respectively) [93] and by Pauli et al., comparing before and after all treatments (9%, 33%, 38%, and 28% at pre-treatment, 3 months, 6 months, and 12 months post-treatment, respectively) [92]. Likewise, Van der Geer et al. reported that prevalence continued to increase over time [98]. Given conflicting study results, it was unclear if sex or disease site had any effect on trismus [91,93,98]. Although no difference in the prevalence of trismus was reported between conventional and accelerated fractionation radiotherapy [93], higher radiation dosage and longer treatment time have been associated with a higher prevalence of trismus [92,98]. Additionally, one study suggested that individuals who drink more than the weekly allowable limit of alcohol were less likely to develop trismus after treatment [91]. In terms of the impact of trismus on patients, Lee et al. found that trismus negatively impacted social contact and social functioning [91].

##### Difficulty Chewing and Dental Problems

Five studies examined the prevalence of chewing difficulties [38,41,42,45,71]. Baseline levels of chewing difficulties were variable among population groups (12–44%). In patients with oral and oropharyngeal cancer, 44% were found to have difficulty chewing at pre-operative assessment [42]. Prior to radiotherapy, Gunn et al. found that 14% (including multiple disease sites) reported difficulty chewing [38]. Within this group, patients with no previous treatment, compared to patients with prior chemotherapy or surgery, had the lowest prevalence (12%, 13%, and 19% respectively) [38]. In comparison, 91% of patients with tongue cancer treated with surgery and radiotherapy reportedly had difficulty chewing an average of 27 months post-treatment [71]. Chewing problems were one of the most prevalent symptoms (98.5%) in patients with nasopharyngeal carcinoma undergoing late-period RT [41]. Six studies evaluated problems with teeth among head and neck cancer patients [5,38,41,45,69,89]. Pre-treatment prevalence ranged from 13% to 27%, while during chemoradiotherapy, prevalence was reported at 82%; at one-year post-treatment, prevalence ranged from 14 to 42% [69,89].

##### Dysgeusia

Fifteen studies evaluated dysgeusia, or loss of taste, among head and neck cancer patients and survivors [5,38,41,42,45,58,71,73,77,79,80,90,99,100,101]. Pre-operative prevalence was found to be 4% [42], while pre-radiotherapy prevalence ranged between 7.5% and 21.5% (for patients with prior chemotherapy and prior surgery, respectively) [38]. Haisfield-Wolfe et al. reported that prevalence increased over the course of treatment with radiotherapy (38%, 86%, and 80% at 1 week, 5 weeks, and 9 weeks, respectively) and dropped to 62% after treatment [58]. Jin et al. reported prevalence of taste alteration of 13% (baseline), 83% (mid-treatment), 92% (post-treatment), and 78% (follow-up) [101]. Using blind taste tests, Baharvand et al. reported a post-radiotherapy prevalence of 100%, with 27% of patients experiencing total taste lost [99]. In a longer-term follow-up study (range 85 days to 28 years), McLaughlin et al. found that 92% of patients still experienced some taste loss [100]. The prevalence of long-term taste loss was even higher (100%) for patients with tumors of the tongue at an average of 27 months post-treatment [71]. As noted previously, loss of taste was also significantly associated with critical weight loss [60,101].

##### Changes in Appetite

The prevalence of decreased appetite was reported among 10 studies [5,30,38,40,41,45,58,69,73,81]. Bond et al. reported that, overall, 96% of their patients reported trouble with appetite [30]. Prior to radiotherapy, Gunn et al. found that patients with previous chemotherapy had a higher prevalence of a lack of appetite (12%) versus those who had undergone previous surgery (5%) or no previous treatment (6%) [38]. During radiotherapy, patients seemed to experience a dramatic increase in a lack of appetite (33%, 91%, and 80% prevalence at 1, 5, and 9 weeks, respectively), dropping to approximately 48% immediate post-radiotherapy [58]. Xiao et al. reported a prevalence of 95% of lack of appetite during (chemo)radiotherapy [41]. Kubrak et al. found a 24% prevalence of loss of appetite at diagnosis [5].

##### Weight Change and Malnutrition

Loss of appetite, loss of taste, and dysphagia are significantly associated with critical weight loss [60]. Sixteen studies reported malnutrition (clinician reported) and weight loss, with prevalence ranging between 3% and 95% [5,34,35,45,56,58,60,62,70,73,81,102,103,104,105,106]. Most studies defined critical weight loss as involuntary loss of more than 5% of normal weight within the past 1 to 6 months [34,56,58,60,62,102,103,104]. Baseline prevalence of malnutrition ranged from 8.5% (at diagnosis) up to 42% (prior to any treatment) [5,34,73,104,105]. During (chemo)radiotherapy, the reported prevalence was much higher (43%, 91%, and 81% at 1 week, 5 weeks, and 9 weeks, respectively) [58]. Although it appears that the prevalence remained high immediately post-treatment, the general trend across studies showed that the prevalence declined over time [70,73,103]. In terms of treatment type, chemotherapy and radiotherapy were significantly associated with greater rates of malnutrition compared to surgery alone and patients treated without chemo or radiation treatment [56,103]. Malnourished patients also experienced worse quality of life compared to adequately nourished patients [102]. Patients indicated a critical need for improved symptom management and/or nutrient intervention options to reduce the burden of weight loss and malnutrition [102].

##### Oral Mucositis

Prevalence of oral mucositis, typically described by patients as presence of mouth or throat sores, was reported in 15 studies, ranging from 44% and 68% prior to treatment [73,74,77,78,79,80,81,88,107,108,109,110,111,112,113]. Elting et al. reported that patients who received chemotherapy had higher prevalence than those without [108]. Simultaneous IMRT caused less oral mucositis compared to conventional treatment (56.0% versus 83.3%, *p* = 0.026) [77]. Arribas et al. reported high prevalence immediately post-treatment (85%), with prevalence declining over time (45% one month after RT and 5% three months after RT) [73]. Thomas et al. reported that subjects who had developed oral mucositis at the end of third week had all progressed to grade 3 or 4 mucositis by the end of therapy [113].

#### 3.3.2. Communication

##### Voice and Speech Impairment

A total of 14 studies examined voice and speech impairment with prevalence ranging from 9% to 88% (Table 2) [21,38,39,41,42,45,55,63,65,66,71,76,114,115]. Among oral and oropharyngeal cancer patients, the pre-treatment prevalence of speech impairment was found to be 42%; however, it is unclear if these patients had undergone any prior treatments [42]. When multiple disease sites were included, the pre-treatment prevalence was found to be much lower at 3% [38]. Post-treatment, the prevalence of voice and speech impairment increased significantly [21,63,65,71,114]. In terms of treatment type, prevalence was found to be higher in patients who received surgery (21.5%) than those who had received chemotherapy (7.5%) or no treatment (3%) [38]. However, when all treatment modalities were compared, patients who received radiotherapy reported the worst functional outcomes for speech [66]. However, in this study, RT was only given to late-stage cancer patients, and thus comparison between treatment types can be biased. In addition, Dwivedi et al. reported that oral cavity patients perceived more problems with voice and speech than oropharyngeal cancer patients [55]. Suarez-Cunqueiro et al. found that patients with tumors located in the floor of the mouth and oropharynx reported worse scores for speech compared to other tumor locations [66]. Only 7 of 14 studies used PROM instruments that were specifically designed to assess voice and/or speech impairment (VHI, VRQOL, GRBAS, SHI), while the rest used generic QOL instruments such as UWQOL, MDASI-HN, and FACT-HN.

##### Hearing Loss

Four studies examined prevalence of hearing loss among HNC patients [45,78,116,117]. In a small cross-sectional study (*n* = 11 patients), Liberman et al. reported that 36% of patients with laryngeal or hypopharyngeal cancer experienced hearing loss; however, the timing of this assessment was unclear [117]. Schultz et al. reported a prevalence of 72% hearing loss more than two years after treatment with radiotherapy in a study involving multiple HNC anatomic subsites [116]. The prevalence of hearing loss in this population was significantly higher than that of an age-matched control group treated with local surgery alone [116]. Huang et al. 2015 reported that IMRT technique was associated with less hearing loss [78].

#### 3.3.3. Pain

Pain was reported in 22 studies with prevalence rates from 9% to 91% (Table 2) [5,21,34,37,38,40,41,42,45,48,58,60,70,77,81,93,118,119,120,121,122,123]. Most studies did not report the type or location of pain, and measurement tools were not consistent. Two studies used a visual analogue scale (VAS) to assess pain [21,119], three studies used MDASI-HN [38,40,41], one used self-reported pain [122], three studies did not describe their method of assessment [118,120,121], two used Common Terminology Criteria for Adverse Events (CTCAE) [77,81], and the remaining studies each used a different assessment tool. One study estimated that as many as 36% of HNC experienced pain at the time of diagnosis [5]. However, during treatment with (chemo)radiotherapy, the prevalence of pain appeared to rise dramatically [41,58,121]. In fact, Pignon et al. reported that 71% of patients in their study experienced pain during radiotherapy and 30% of those patients were experiencing “new pain”, most likely caused by treatment [120]. Post-treatment, a general trend towards decreasing prevalence of pain was noted over time [70,121]. Two studies examined risk factors for pain, finding that, in general, a higher cancer stage was associated with increased levels of pain [48], while gender, treatment modality, and tumor site were not [119]. Cramer et al. identified that tri-modality treatment with surgery with adjuvant chemoradiation was the only characteristic associated with pain [122]. Pain was consistently listed as one the most distressing symptoms at each measurement period among studies [42,58,63].

#### 3.3.4. Dyspnea and Cough

Three studies reported the prevalence of dyspnea or shortness of breath (Table 2) [37,38,41]. Baseline levels of dyspnea were estimated at 6% in this population [38], while Lokker et al. estimated that approximately 21% of HNC in the palliative phase of care experienced dyspnea [37]. The prevalence of dyspnea in palliative patients was highest in those treated with chemotherapy (12%) compared to surgery alone (4%) or other treatments (3%) [58]. During (chemo)radiotherapy, the prevalence of dyspnea was reported at 68% [41]. Three studies examined the prevalence of cough, which ranged between 10.5% and 52% (Table 2) [69,70,124]. Prior to treatment, Ginex et al. found a prevalence of 32% in esophageal cancer patients [70]. This same study found that symptoms of cough worsen post-operatively but recovered to baseline at one year. The prevalence of cough seemed to be independent of early versus late tumor stage [69].

### 3.4. Functional Well-Being

Some studies evaluated prevalence of functional well-being in HNC (Table 3).

#### 3.4.1. Activities of Daily Living

##### Difficulties with Activities of Daily Living

Prior to treatment, Lango et al. reported that 9% of patients had problems with mobility, 2% with self-care, and 14% with performing usual activities [34]. As no reference population was used to compare these results, it is difficult to assess the severity of these symptoms (Table 3).

##### Sexual Function

Problems with sexual function were reported in two studies (Table 3) [20,39]. In one study, 32% of patients reported that they were less interested in sex following a laryngectomy, while 42% of males had erectile dysfunction [20]. The same study concluded that sexual problems were not treatment-related but were likely caused by the cancer itself [20]. Distress and depression were strongly correlated with sexual difficulties (*p* < 0.01) [20]. Beyond prevalence data, Ginex et al. found that patients reported problems with sexual activity and interest as one of the most bothersome symptoms both pre- and post-surgery [70]^.^

#### 3.4.2. Fatigue and Energy

##### Fatigue

The prevalence of fatigue, or decreased energy, was reported in 14 studies, ranging from 7% to 95% (Table 3) [23,37,38,39,40,41,45,58,69,70,77,123,126,127]. The baseline prevalence of fatigue prior to any treatment ranged from 14.5% to 58% [38,70]. The prevalence of fatigue appeared to increase over the course of treatment with radiotherapy (71%, 91%, and 95% at 1 week, 5 weeks, and 9 weeks, respectively) [58]. However, post-radiotherapy, prevalence was likely to return to baseline levels [58]. A different picture is shown post-surgery, as the prevalence of fatigue was worse immediately after surgery but recovered to baseline by one year [70]. In a study by Qian et al., all patients reported some level of fatigue; however, patients considered mild fatigue to be normal, while 13% reported moderate fatigue [126]. McDowell et al. reported prevalence of moderate (14%) and severe (14%) fatigue even four years after treatment [23].

##### Decreased Alertness/Drowsiness

In one study, drowsiness was reported by 11% patients prior to treatment with radiotherapy [38] and by 22% of treatment-naïve patients [40]. During (chemo)radiotherapy, 91% of patients with nasopharyngeal carcinoma experienced drowsiness [41]. After treatment, 70% of caregivers reported that patients experienced decreased alertness (Table 3) [30].

##### Sleep Quality 

The prevalence of difficulty sleeping or sleep disturbance ranged from 16% to 100% across 11 studies (Table 3) [37,38,40,41,45,70,77,123,126,128,129]. Only one study reported prevalence before and after treatment, finding a bell-shaped trend over time (41%, 62%, and 42% at pre-surgery, immediately post-surgery, and 6 months, respectively) [70]. Qian et al. found a higher prevalence of obstructive sleep apnea in a group of patients treated without surgery (100%) compared to patients treated with surgery (93%), although the surgery group reported more severe symptoms [126]. Li et al. reported a high prevalence of poor sleep quality in long-term HNC survivors [129].

### 3.5. Characteristics of Outcome Measurement Instruments

Among 53 instruments identified by Ojo et al. [12], 45 instruments were reviewed, and information about their PROM items was extracted, resulting in 124 different symptoms identified. Among instruments, 22 instruments assessed general symptoms of HNC and quality of life, 10 assessed eating ability including symptoms such as dysphagia and xerostomia, 6 instruments assessed speech and voice, 2 instruments assessed neck and shoulder disabilities, 3 instruments assessed oral mucositis, and 1 instrument assessed skin symptoms and sinonasal outcomes.

**Figure 3:**
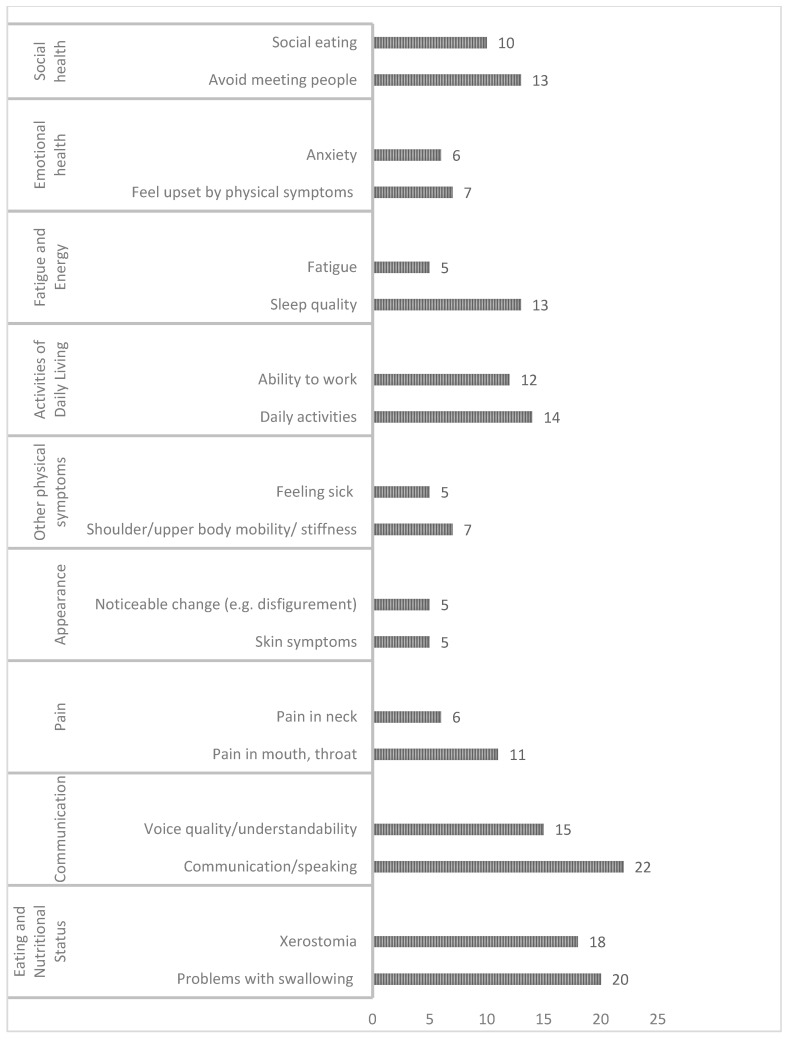
Number of instruments (of 45 assessed) that included items in specific domains. The domains are listed in the left column, the items are listed to the right, and the number of instruments is shown in graphical bars to the right. Not all items are listed in this summary.

Symptoms assessed by each instrument were mapped by the following domains and compared on content: (1) physical symptoms, (2) psychological symptoms, (3) psychosocial symptoms, (4) functional symptoms, and (5) quality of life (Figure 3). The complete cross-comparison of instruments can be found in Supplementary Table S2.

We found major discrepancies between the symptoms reported in the prevalence review and the symptoms captured by the PROMs. While some instruments had comprehensive overlap with the symptoms identified in the prevalence review, a number of the symptoms that recurrently appeared in the PROM instruments were not widely reported in the included studies. For example, 12 different PROM instruments in this review could assess ‘cough’, yet we found only three studies reporting this symptom [69,70,124]. Likewise, we found 15 PROM instruments that assessed ‘changes in appearance’ and its psychological impact, yet only four studies reported this symptom [45,52,53,54]. This discrepancy is even more noticeable in the psychological symptom category. Functional well-being such as performing activities of daily living is broadly covered by 24 instruments, but we found only one study [34] that reported related symptoms. Social and family well-being is covered by 24 instruments in various aspects such as interference with family life or relationship with friends, ability to participate in social activities, and anxiety about social life. However, we found only three studies that examined ‘avoidance of social contacts’ only in relation to this problem category [53,55,56]. Prevalence data may be instrumental for identifying symptom burden in head and neck populations, but their capture of symptoms may be limited by the domains and items in the outcome measures used. On the other hand, PROMs may generate items on the basis of input of clinicians and patients regarding the relevant symptoms in the HNC population in their initial development and content validation process. In selecting PROM measures for routine surveillance in HNC populations, one should consider data from prevalence studies and PROMs for relevant capture of burdensome symptoms.

In summary, on the basis of the prevalence of symptom burden, PROMs for routine surveillance in HNC populations should capture physical well-being domains for eating and weight changes (especially dysphagia, xerostomia, dysgeusia, and weight loss), communication (voice/speech), pain, and fatigue. Depression and anxiety should also be key items in the psychosocial domain of PROMs given its prevalence in HNC. Specific capture of these symptom domains in PROM items could help to identity the impact of HNC and its treatment, thus enabling personalized tailoring of symptom management [130].

On the basis of a cross comparison of symptoms identified in the literature and symptoms addressed in the PROMs (Supplementary Table S1), we identified seven instruments for further review: FACT-NP, FSH&N-SR, HNRT-Q, MDASI-HN, OMQOL, the QOL-Rathmell, and the QOL-Thyroid. These seven instruments were selected for further review as they were frequently used in the prevalence studies, were specific to HNC populations, and covered common physical and emotional HNC symptoms that our expert team members considered important for routine surveillance in HNC populations. Content domains and number of the items from each PROMs are displayed in Table 4.

### 3.6. Psychometric Comparison of the Instruments

Searches for studies evaluating the validity of these instruments were conducted, and seven validation studies of PROM were identified [131,132,133,134,135,136,137]. No validation studies of QLQ-Rathmell and QOL-Thyroid were identified.

The quality of these studies were assessed using the COSMIN checklist, which provides an overall rating based on the quality of each article assessing internal consistency, reliability, measurement error, content validity, structural validity, hypothesis testing, criterion validity, responsiveness, and interpretability [138]. Articles evaluating or describing the translation of these PROs into languages other than English were not evaluated in this review. Results of our assessments of these validation studies are provided in Supplementary Table S3.

Table 5 shows the psychometric properties that were reported in the included studies. Internal consistency was reported in all studies and for all the tools, yet no studies assessed measurement error or interpretability of the tools. Test–retest reliability and convergent validity were reported in two studies each [131,132,133,134]. Known-groups validity, concurrent validity, and responsiveness were reported for three tools [132,134,135], and content validity was also reported for one tool [133]. As seen in Table 5, the OMQOL was evaluated by seven properties, while assessment of other tools was conducted on the basis of three or four properties. For HNRT-Q, only internal consistency was reported.

#### 3.6.1. Reliability

All of the tools demonstrated high Cronbach’s alpha (α) (0.84–0.97). FACT-NP, OMQOL, and HNRT-Q showed excellent level of alpha for the total items (α ≥ 0.9) [131,133,137]. Among these tools, OMQOL demonstrated the highest α for both subscales and the total items [133]. Test–retest reliabilities were reported for the FACT-NP and the OMQOL. Both tools demonstrated good test–retest reliabilities, yet the OMQOL demonstrated the higher intraclass correlation coefficient (ICC) on the subscales (0.864–0.934) [133]. No studies reported measurement errors for assessing reliability of the tools.

#### 3.6.2. Construct Validity (Convergent Validity, Known-Groups Validity)

Three studies reported construct validity but using different methods. While Rosenthal et al. used known-groups validity for evaluating MDASI-HN [135], Baker et al. and Cheng et al. assessed convergent validity and known-groups validity for the FSH&N-SR and the OMQOL, respectively [132,134].

These studies showed variation in the measurement of convergent and known-groups validity. Baker et al. computed Pearson’s correlation coefficients between the FSH&N-SR and the Karnofsky Performance Scale (KPS), the 36-Item Short Form Survey (SF-36), and the Performance Status Scale for HNC patients [132]. On the other hand, Cheng et al. calculated Pearson’s correlation coefficient for correlations between the OMQOL subscales and OM (oropharyngeal mucositis)-related symptoms peak and AUC (area-under-the-curve) scores [134].

For known-groups validity measurement, Baker et al. used *t*-test for two different patient groups [132], whereas Cheng et al. compared the OMQOL subscales peak and AUC scores among patients with different levels of OM and types of cancer therapy [134]. Rosenthal et al. compared mean scores of MDASI subscales between the patient groups categorized into good and poor performance status [135].

Given the variability in measurement approaches, direct comparisons are impossible; there is no way to conclude that any one instrument has shown better construct validity than another.

#### 3.6.3. Criterion Validity (Concurrent Validity)

Criterion validities of FACT-NP, the OMQOL, and the MDASI-H&N were confirmed by assessing concurrent validities. Again, the measurement methods varied across studies. Tong et al. computed Pearson’s correlation coefficients between the subscales of FACT-NP and those of the QOL-RTI-H&N [131]. Moderate or high correlations were found, which indicated concurrent validity of FACT-NP. Cheng et al. assessed Pearson’s correlation coefficients between the OMQOL subscales peak and AUC scores with those of EORTC [134]. Moderate correlations confirmed the concurrent validity of the OMQOL. Weak or moderate correlations were found between the subscales of the MDASI-H&N and the 12-item Short-Form Health Survey (SF12v2), yet the study concluded that concurrent validity had been confirmed. MDASI scores were significant predictors of objective CTCAE scores on multivariate regression analysis [136].

#### 3.6.4. Responsiveness

Responsiveness was confirmed for the FACT-NP, the FSH&N-SR, and the OMQOL. Tong et al. and Cheng et al. used effect size comparisons and confirmed that the FACT-NP and the OMQOL were responsive to the changes in the scores over time [131,134]. Baker et al. found that the FSH&N-SR demonstrated responsiveness to changes by cancer stage and the extent of initial surgical procedure using ANOVA and pairwise comparisons [132].

## 4. Discussion

In this review, we identified symptoms experienced by HNC populations, described their prevalence, and identified HNC-specific PROMs and their coverage of the physical and emotional symptom problems experienced by this population.

The prevalence of reported symptoms was highly variable among included studies. Variability in sample size, the timing of the assessments, and the use of different measures may explain some of this variability. HNC patients experience symptoms common to many other cancer patients but can also experience disease-specific or treatment-specific symptoms (i.e., dysphagia); evaluating both types of symptoms will be important to understand the burden of disease and treatment in this population.

The PROMs used varied across studies, thus precluding meta-analysis for estimating prevalence of symptoms. For example, depression was assessed using the Hospital Anxiety and Depression Scale (HADS) [16,17,18,19,20,21,22,23,24,25], the Beck Depression Inventory (BDI) [16,26,27,28], the short-form of the Geriatric Depression Scale (GDS-SF) [29], the Neuropsychiatric Inventory Questionnaire (NPI-Q) [30], the Research Diagnostic Criteria Schedule for Affective Disorders and Schizophrenia (RDC SADS) [31], and the University of Washington Quality of Life Mood scale (UWQOL-mood) [32]. Furthermore, there was variability in cut-off scores used for the same instrument. For example, thresholds for HADS ranged from 7 to 11 and BDI thresholds ranged from 10 and 21. There is a need for standardization in PROM items for use in patient management for routine care and population comparison. A recent review recommended the Patient Health Questionairre-9, Zung Self-Rating Depression Scale, and Zung Self-Rating Anxiety Scale as having good content coverage and excellent psychometric properties to assess psychological distress in HNC populations [139].

The symptoms and their prevalence experienced by HNC patients varied widely, depending on the cancer site, treatment modalities, and phase of treatment. Thus, choice of PROM should focus on the content and its temporal application relative to the phases of the cancer journey (pre-treatment, during treatment, after treatment, during surveillance, etc.). Standardization in the temporal application of PROMs is also needed. We recommend that studies consider measuring depression, pain, dysphagia, and dysgeusia outcomes especially during treatment, in which the highest prevalence was noted. The following time points—during treatment, after treatment, during surveillance—should be considered when measuring symptoms that worsen during treatment and remain at higher levels into follow-up (e.g., trismus, xerostomia, and speech difficulties). However, many of these symptoms can persist as long-term problems post-treatment.

Standardization in the criteria used for validation of PROMs is also crucial, given wide variability across studies. For example, validation of FACT-NP was based on criterion validity and responsiveness [131], while FSH&N-SR was validated on the basis of construct validity and responsiveness [132]. The MDASI-H&N was evaluated by both construct validity and criterion validity [135]. Furthermore, the measurement methods for the same psychometric property were also highly variable. For example, Baker et al. and Cheng et al. assessed both convergent validity and known-groups validity to evaluate construct validity of the tools [132,134], while Rosenthal et al. only assessed known-groups validity [135]. For known-groups validity, Baker et al. conducted a *t*-test for the FSH&N-SR [132], and Rosenthal et al. compared mean scores of the MDASI subscales between two patient groups [135]. On the other hand, Cheng et al. compared OMQOL subscales peak and AUC scores between different OM severities groups [134]. Similarly, variability was found in the assessment methods for concurrent validity and responsiveness. Due to this variability, it is difficult to make meaningful comparison across measures in terms of psychometric properties. We can only conclude that there is at least some evidence supporting the validity of the PROM instruments; thus, the psychometric properties and content of multiple PROM instruments should be considered before selection and depending on purpose, i.e., routine surveillance and or research. Moreover, it is essential to carefully consider the content of each PROM before choosing it [140].

In order to determine the optimal choice of tools for monitoring symptoms in HNC patients, from the 45 instruments, on the basis of a cross comparison of symptoms identified in the literature and symptoms addressed in the PROMs, we were able to identify seven instruments for further review. Our findings do not suggest that the other PROMs are unacceptable as instruments to capture symptom burden in patients with HNC. However, a combination of different PROMs may be necessary to ensure capture of the important domains. We recommend further validation studies of the identified PROMs, as well as development of HNC-specific PROMs, in order to foster personalized symptom management, and to reduce survey fatigue.

There are limitations to our study. We only included studies from the last 15 years, restricted to the English-language. As such, the prevalence of some symptoms may be under or over-represented in our review. We did not restrict our analysis by methods used to assess the various symptoms. Therefore, there was wide variability in both the assessment and the definition of various symptoms. This was also reflected in the wide variability in symptom prevalence across studies. Given the heterogeneity of measurement tools and threshold values used, meta-analysis could not be performed on our group of studies, and we could not report on a final estimated prevalence for each of the symptoms. Quality of the prevalence studies was subjectively determined by reviewers and not used for exclusion purposes. Therefore, some caution should be applied when interpreting the findings of the report.

## 5. Conclusions

Our search identified wide variability in the specific symptoms assessed and their prevalence and in the content and psychometric validity of measurement tools. Further, there was some discrepancy between the symptoms reported in the included studies and the retrieved PROMs, suggesting incomplete reporting of important HNC symptoms and problems and potential for underestimation of impact. We recommend that journals either require or strongly recommend that authors provide public access to the raw and complete data from PROMs studies, which would help promote transparency, meta-analysis, and pooled analysis of data. Thus, we recommend standardization of elements such as inclusion of certain treatment- and condition-specific PROM items, as well as standardization of temporal application of PROMS relative to key events such as treatment or disease relapse, in order to promote cross-collaboration and cross-comparison across studies. Either the FACT-HN or the MDASI could be used in routine surveillance as they provided the most complete coverage of prevalent physical and emotional symptoms and had adequate psychometric properties, but supplementation with condition specific measures (i.e., dysphagia, body image disturbance) may be needed depending on the purpose of measurement.

## Figures and Tables

**Table 1:** Prevalence of psychosocial symptoms and emotional well-being in head and neck cancer patients.

Symptom	Cancer Type	Treatment	Measure and Cut-Off Score	Number of Studies	Range of Prevalence				
					Pre-Treatment	Treatment	Post-Treatment	Overall/ NS *	References
Depression	Oral cavity, larynx, Oropharynx, salivary gland, nasal cavity, thyroid, nasopharynx, unknown primary, paranasal sinus	Surgery ± RT ± chemo	SADS (RDC criteria), BDI, HADS-D, UWQOL-mood, NPI-Q, GDS-SF, PHQ-8	22	7.5–84%	7–75%	2–78%	46%	[4,16,17,18,19,20,21,22,23,24,25,26,27,28,29,30,31,32,33,34,35,36]
Sadness	Oropharynx, oral cavity, larynx, nasopharynx, skin, hypopharynx, skull base, thyroid, nasal cavity/sinus, salivary gland	RT ± chemo ± surgery or treatment-naive patients	MDASI-HN, Pal-C, FACT-HN, Not specified	5	8–27%	82%		19–57%	[37,38,39,40,41]
Anxiety	Oropharynx, thyroid, oral cavity, larynx, parotid gland, paranasal sinus, nasopharynx salivary gland (not specified)	RT ± chemo ± surgery or treatment-naive patients	HADS-A, UWQOL, GAD-2, Pal-SI, NPI-Q, PCI, Not specified,	20	20–72%	34.5%	1–97.5%	12–29%	[4,16,17,18,19,20,21,22,23,25,30,33,35,36,37,42,43,44,45,46]
Worry	Oral cavity, sinus, oropharynx, larynx, pharynx, salivary gland, nasal fossa	Surgery ± RT ± chemo	MSAS, Pal-C, NPI-Q	3	38–62%	30–57%	33–52%	61%	[30,37,47]
Distress	Oropharynx, oral cavity, larynx, salivary gland, nasopharynx, sinus, hypopharynx, skin, thyroid, skull base, nasal cavity, neck,	RT ± chemo ± surgery or treatment-naive patients	MDASI-HN, DT	7	14–51%	86%	33–35%	44.5%	[38,40,41,48,49,50,51]
Satisfaction with Appearance	Oral cavity, skin, mid-face, larynx, oropharynx, hypopharynx, nasopharynx, unknown (not specified)	Surgery ± chemo or ±RT	MBSRQ, BASS, BIS, PCI	4	11%	25–27%	73–75%	89%	[45,52,53,54]
Avoidance of Social Interactions	Oral cavity, oropharynx, skin, cancer of the mid-face, maxilla cancer, others	Surgery ± RT ± chemo	Speech Handicap Index BIS	3	-	-	16–62%	-	[53,55,56]

* Overall/NS: prevalence was reported but timing of assessment was not specified. RDC-SADS = Research Diagnostic Criteria Schedule for Affective Disorders and Schizophrenia, BDI = Depression Inventory, GDS-SF = Geriatric Depression Scale—short-form, NPI-Q = Neuropsychiatric Inventory Questionnaire, UWQOL-mood = University of Washington Quality of Life Mood scale, PHQ-8 = Patient Health Questionnaire 8, MDASI-HN = MD Anderson Symptom Inventory—Head and Neck, PAL-C = Palliative Checklist, FACT-HN = Functional Assessment of Cancer Therapy—Head and Neck, HADS-A = Hospital Anxiety and Depression Scale, GAD-2 = Generalized Anxiety Disorder Questionnaire 2, Pal-SI = Palliative Symptom Impact list, PCI = Patient Concern Inventory, MSAS = memorial symptom assessment scale, DT = Distress Thermometer, MBSRQ = Multidimensional Body-Self Relations Questionnaire, BASS = Body Areas Satisfaction Scale, BIS = Body Image Scale.

**Table 2:** Prevalence of physical symptoms in head and neck cancer patients.

Symptom	Cancer Type	Treatment	Measure and Cut-Off Score	Number of Studies	Range of Prevalence				References
					Pre-Treatment	Treatment	Post-Treatment	Overall/ NS *	
Eating And Weight Changes									
Dysphagia	Oral cavity, larynx, oropharynx, thyroid hypopharynx, skin, nasopharynx, sinus pharynx, nasal fossa salivary gland, nasal cavity, primary maxillary, unknown	Surgery ± RT ± chemo Or not specified	SWAL-QOL, MDADI, MDASI-HN, PCI, PG-SGA, EAT-10, Pal-C, CTCAE, UW-QOL, MSAS-SF, QLQ H&N-35, FACT-HN, FEES, Not specified, medical record, chest X-ray	35	12–75%	38–100%	0–100%	28–80%	[4,5,37,38,39,41,42,45,56,58,60,61,62,63,64,65,66,67,68,69,70,71,72,73,74,75,76,77,78,79,80,81,82,83,84]
Xerostomia	Oral cavity, naso/oropharynx, skin, hypopharynx, larynx, salivary glands, thyroid, nasal cavity/sinus, unknown, skull base	Surgery ± RT ± chemo or not specified	PCI, UW-QOL, EORTC QLQ-C30/H&N-35, FACT-HN, PG-SGA, MFIQ, CTCAE, MSAS, study-specific questionnaire	23	4–18%	71–97.5%	0–100%	36–80%	[5,38,39,40,41,42,45,58,65,67,69,71,73,77,78,79,80,85,86,87,88,89,90]
Trismus	Oral, oropharynx, larynx, neck, ear hypo/nasopharynx, salivary/parotid gland, thyroid, sinus salivary gland, unknown	RT ± chemo ± surgery or treatment-naive	MIO, EORTC H&N35, EORTC QLQ-C30, MFIQ, PCI, CTCAE	14	3–41%	-	12–57%	4–19%	[24,45,69,77,86,90,91,92,93,94,95,96,97,98]
Difficulty chewing	Oropharynx, oral cavity, nasal cavity/sinus, salivary gland hypo/nasopharynx, larynx, thyroid, skin, unknown	RT ± chemo ± surgery (prior tx not described)	UW-QOL MDASI-HN, PCI, CTCAE	5	12–44%	98.5%	91%	30%	[38,41,42,45,71]
Dysgeusia/ Taste	Oropharynx, oral cavity, naso/hypopharynx, larynx, thyroid, salivary gland, nasal cavity/sinus, skin, maxilla/mandible parotid unknown	RT ± chemo ± surgery (prior tx not described)	MSAS, UW-QOL, MDASI-HN, pipette droplet, EORTC QLQ H&N35, PCI, PG-SGA, STA	15	3–21.5%	38–97%	1–100%	27–76%	[5,38,41,42,45,58,71,73,77,79,80,90,99,100,101]
Dental problems	Oropharynx, skin, oral cavity, larynx, salivary gland naso/hypopharynx, nasal cavity/sinus, thyroid, unknown	RT ± chemo ± surgery	EORTC QLQ-C30, MDASI-HN, PCI, PG-SGA	6	13–27%	82%	14–42%	19%	[5,38,41,45,69,89]
Malnutrition/weight loss	Oropharynx, oral cavity, esophageal naso/hypopharynx, larynx, maxillary sinus, submandibular gland unknown	Chemo ± surgery ± RT or none Not specified	BMI, albumin, weight loss, MSAS, MSAS-SF, PCI, PG-SGA, FFMI, WLG, hand grip	16	8.5–42%	43–91%	3–95%	17%	[5,34,35,45,56,58,60,62,70,73,81,102,103,104,105,106]
Lack of appetite	Oropharynx, oral cavity, skull base salivary gland, skin, hypo/nasopharynx, larynx, thyroid, nasal cavity/sinus, maxilla, others unknown	RT ± chemo ± surgery treatment-naive	MSAS, EORTC QLQ-C30, NPI-Q, MDASI-HN, PCI, PG-SGA, CTCAE	10	5–24%	33–95%	20.0–48.0%	22–96%	[5,30,38,40,41,45,58,69,73,81]
Oral mucositis	Oral cavity, larynx oropharynx, hypo/nasopharynx, Not specified others	RT ± chemo ± surgery	CTCAE, OMDQ PG-SGA, WHO grading, not specified	15	44–68%	7–100%	2–85%	42–83%	[73,74,77,78,79,80,81,88,107,108,109,110,111,112,113]
Communication									
Voice/speech	Oropharynx, oral cavity, maxillary naso/hypopharynx, larynx, thyroid, salivary gland, nasal cavity/sinus, skin unknown	RT ± chemo ± surgery Not specified	UW-QOL, VHI, MDASI-HN, FACT-HN, PCI DÖSAK, SHI V-RQOL, GRBAS	14	3–55%	9–85%	20–91%	16–64%	[21,38,39,41,42,45,55,63,65,66,71,76,114,115]
Hearing loss	Larynx, naso/hypopharynx, parotid, oral cancer, unknown	RT + chemo, surgery, surgery + RT	PCI, CTCAE not specified	4	-	-	2–72%	18%	[45,78,116,117]
Pain									
Pain	Oropharynx, oral cavity, thyroid, nasopharynx, larynx, esophageal hypopharynx, salivary gland, nasal cavity/sinus, skin visceral, parotid, neck SCC unknown skull base	RT ± chemo ± surgery Not described treatment-naive patients	NRS, Pal-C, MDASI-HN, MSAS, VAS, UW-QOL, PCI, UMCG H&N CST, EORTC H&N35, PG-SGA, VHNSS, Self-report pain, EQ5D-3L CTCAE, not specified	22	9–50%	62–89%	31–91%	20–54%	[5,21,34,37,38,40,41,42,45,48,58,60,70,77,81,93,118,119,120,121,122,123]
Other Physical Symptoms									
Dyspnea	Oropharynx, oral cavity, larynx, hypo/nasopharynx, thyroid/trachea, salivary gland, nasal cavity/sinus, skin	RT ± chemo ± surgery	Pal-C, MDASI-HN	3	3–12%	68%	-	21%	[37,38,41]
Cough	Esophageal oral cavity, oropharynx, hypo/nasopharynx, larynx, maxilla	RT ± chemo ± surgery	EORTC QLQ-C30 MSAS-SF, Pearson’s scale	3	32%	-	10.5–52%	-	[69,70,124]

* Overall/NS: prevalence was reported but timing of assessment was not specified. SWAL-QOL = Swallowing Quality of Life questionnaire; MDASI-HN = MD Anderson Symptom Inventory—Head and Neck; PCI = Patient Concern Inventory; PG-SGA = Patient-Generated Subjective Global Assessment; EAT-10 = Eating Assessment Tool; PAL-C = Palliative Checklist; CTCAE = Common Terminology Criteria for Adverse Events; UW-QOL = University of Washington Quality of Life Questionnaire; MSAS-SF = Memorial Symptom Assessment Scale–Short Form; EORTC QLQ-H&N35 = European Organization for the Research and Treatment of Cancer Quality of Life Questionnaire—head and neck cancer-specific module; FACT-HN = Functional Assessment of Cancer Therapy—Head and Neck; FEES = flexible endoscopic evaluation of swallowing; MFIQ = mandibular function impairment questionnaire; MSAS = memorial symptom assessment scale; MIO/MID = Maximal Interincisal Distance/Opening; STA: subjective taste alteration; BMI = body mass index; WLG = weight loss grade; FFMI = fat-free mass index; EORTC QLQ-C30 = European Organization for the Research and Treatment of Cancer Quality of Life Questionnaire; NPI-Q = Neuropsychiatric Inventory Questionnaire; OMDQ = Oral Mucositis Daily Questionnaire; WHO grading = World Health Organization grading; VHI = Voice Handicap Index; DÖSAK = *Deutsch-Österreichisch*-Schweizerischer Arbeitskreis für Tumoren im Kiefer und Gesichtsbereich rehabilitation questionnaire; SHI = Speech Handicap Index V-RQOL = Voice-Related Quality of Life; GRBAS = Grade, Roughness, Breathiness, Asthenia, Strain scale; NRS = Numerical Rating Scale; VAS = Visual Analogue Scale; UMCG H&N CST = University Medical Center Groningen Head and Neck Clinical Screening Tool; VHNSS = Vanderbilt Head and Neck Symptom Survey; EQ-5D-3L = EuroQol Group Questionnaire; MDADI = M. D. Anderson dysphagia inventory.

**Table 3:** Prevalence of functional problems in head and neck cancer patients.

	Cancer Type	Treatment	Measure and Cut-Off Score	Number of Studies	Range of Prevalence				References
					Pre-Treatment	Treatment	Post-Treatment	Overall/ NS *	
Activities of Daily Living									
Activities of daily living	Oral cavity, oropharynx, larynx, naso/hypopharynx, unknown	NR	EQ-5D-3L	1	2–14%	-	-	-	[34]
Sexual function	Larynx, hypo/pharynx oral cancer, salivary glands	Surgery ± RT or NR	EORTC QLQ-H&N35, FACT-HN	2	-	-	42%	32–42%	[20,39]
Fatigue and Energy									
Fatigue	Oropharynx, oral cavity, hypo/nasopharynx, larynx, pharynx, thyroid, salivary gland, nasal cavity/sinus, skin maxilla unknown skull base	Surgery ± RT ± chemo Not reported	MSAS, Pal-C, MDASI-HN, EORTC QLQ-C30, ESS, MSAS-SF FACT-HN, PCI, BFI, VHNSS, CTCAE	14	14–58%	71–95%	7–85%	7–81%	[23,37,38,39,40,41,45,58,69,70,77,123,126,127]
Drowsiness/ decreased alertness	Oropharynx, oral cavity, larynx, skin hypo/nasopharynx, thyroid, salivary gland, nasal cavity/sinus, skull base	RT ± chemo ± surgery or treatment-naïve	MDASI-HN, NPI-Q	4	8–22%	91%	70%	-	[30,38,40,41]
Sleeping problems	Oropharynx, oral cavity, larynx, hypo/nasopharynx, thyroid, salivary gland, nasal cavity/sinus, skin, skull base, esophageal unknown, not described	Surgery ± RT ± chemo or treatment-naïve	Pal-SI, MDASI-HN, MSAS-SF, RDI, PCI, AHI, PSQI, VHNSS, CTCAE	11	16–41%	94.5%	40–100%	0–29%	[37,38,40,41,45,70,77,123,126,128,129]

* Overall/NS: prevalence was reported but timing of assessment was not specified. MDASI-HN = MD Anderson Symptom Inventory—Head and Neck, PCI = Patient Concern Inventory, PAL-C = Palliative Checklist, CTCAE = Common Terminology Criteria for Adverse Events, MSAS-SF = Memorial Symptom Assessment Scale—Short Form, FACT-HN = Functional Assessment of Cancer Therapy—Head and Neck, EORTC QLQ-C30 = European Organization for the Research and Treatment of Cancer Quality of Life Questionnaire, NPI-Q = Neuropsychiatric Inventory Questionnaire, VHNSS = Vanderbilt Head and Neck Symptom Survey, BFI = Brief Fatigue Inventory, EQ-5D-3L = EuroQol Group Questionnaire, EORTC QLQ-H&N35 = European Organization for the Research and Treatment of Cancer Quality of Life Questionnaire—head and neck cancer-specific module, MSAS = memorial symptom assessment scale, ESS = Epworth Sleepiness Scale, Pal-SI = Palliative Symptom Impact list, RDI = Respiratory Disturbance Index, AHI = Apnea–Hypopnea Index, PSQI = Pittsburgh Sleep Quality Index.

**Table 4:** Coverage of symptoms: number of items related to each symptom in selected PROMs.

PROMS Domain		Symptoms	FACT-NP	FSH&N-SR	HNRT-Q	MDASI-HN	OMQOL	QLQ-Rathmell	QOL-Thyroid
Disease Specific									
Physical well-being	Eating and weight changes	Swallowing problems (e.g., swallowing different type of food, painfulness, stressfulness, etc.)	1	1	1	1	3	1	-
		Saliva/dry mouth/drooling (xerostomia)	1	2	2	1	1	2	-
		Cough/chocking when swallowing	-	-	-	1	1	-	-
		Trismus	-	-	-	-	1	-	-
		Mucus/phlegm	-	-	-	1	-	-	-
		Appetite/eating/taste(chewing, teeth/dentures/gum problem, taste/smell, eating speed, ability of eating, use of nutritional supplements/stomach tube, change in diet and quantity of food intake)	3	3	5	4	7	3	1
		Weight change	-	-	-	-	1	1	1
	Communication	Voice change	2	1	1	1	1	1	1
		Communication/talking/speaking	1	1	-	-	5	1	-
		hearing loss	2	-	-	-	-	-	-
	Appearance	Noticeable change (e.g., disfigurement)	1	1	-	-	-	2	1
		Ulceration/erythema (oral, cheek)	-	-	-	-	1	-	-
		Skin symptoms	-	-	2	1	-	-	1
	Pain	Pain	2	2	2	3	5	1	1
	Fatigue and energy	Sleep issue (drowsy, sleep quality)	1	-	1	2	1	-	1
		Fatigue	1	1	3	1	-	1	1
		Breathing	-	1	-	1	-	-	-
	Other physical symptoms	Feeling sick	1	-	-	-	-	1	-
		Nausea/upset stomach/vomiting	1	-	2	1	-	2	-
		Loss of vision	1	-	-	-	-	-	-
		Shoulder/upper body mobility/stiffness	1	1	-	-	-	-	-
		Constipation	-	-	-	1	-	-	1
		Swelling in mouth	-	-	-	-	1	-	-
		Memory problem	-	-	-	1	-	-	1
		Tolerance to cold or heat	-	-	-	-	-	-	1
		Swelling/fluid retention	-	-	-	-	-	-	1
		Menstrual changes or fertility	-	-	-	-	-	-	1
		Nasal outcomes (sneezing, runny nose, nasal discharge)	1	-	-	-	-	-	-
		Motor skills/coordination	-	-	-	-	-	-	1
		Throat discomfort	-	-	-	-	1	-	-
Generic									
Quality of life (general)/rate overall quality of life			1	1	-	-	-	-	1
Overall health			-	-	-	-	-	-	1
Functional well-being		Physical function (ability to work, daily activities, ability to walk, drive, concentrate, to engage in recreational activities)	2	-	2	3	-	1	9
		Enjoyment of food (includes ability to eat favorite food)	1	-	-	-	1	-	-
		Enjoyment of life	1	-	-	1	-	-	-
		Enjoyment of things for fun	1	-	-	-	-	-	-
		Income loss/financial burden	-	-	-	-	-	-	1
		Sexual enjoyment	1	-	-	-	-	-	1
Psychological/emotional well-being		Psychological distress (distress, bothered, upset, unhappy with symptoms, appearance, treatment, uncertainty etc.)	3	-	-	1	1	-	8
		Life satisfaction	1	1	-	-	-	-	1
		Emotional function	7	1	2	1	-	1	12
		Spiritual life	-	-	-	-	-	-	2
Social well-being		Social function	3	1	2	1	2	2	2
		Acceptance of illness by family	2	-	-	-	-	-	-
		Family communication about illness	1	-	-	-	-	-	-
		Support from family/friends	2	-	-	-	-	-	1

MDASI-HN = MD Anderson Symptom Inventory—Head and Neck, FACT-NP = Functional Assessment of Cancer Therapy—Nasopharyngeal module, HNRT-Q = Head and Neck Radiotherapy Questionnaire, MDASI-HN = M.D. Anderson Symptom Inventory—Head and Neck, QOL = quality of life, OMQOL = Oral Mucositis Quality of Life Measure, FSH&N-SR = Functional Status in Head and Neck Cancer—Self Report.

**Table 5:** Psychometric properties reported for the PROMs.

Instruments	Internal Consistency	Test–Retest Reliability	Measurement Error	Content Validity	Construct Validity		Criterion Validity	Responsiveness	Interpret-Ability
					Convergent Validity	Known Groups Validity	Concurrent Validity		
FACT-NP [131]	X	X					X	X	
FSH&N-SR [132]	X				X	X		X	
OMQOL [134]	X	X		X	X	X	X	X	
MDASI-H&N [135,136]	X					X	X		
HNRT-Q [137]	X								
QOL-Rathmell	There were no validation studies for these instruments								
QOL-Thyroid									

FACT-NP = Functional Assessment of Cancer Therapy-Nasopharyngeal, FSH&N-SR = Functional Status in Head and Neck Cancer—Self Report, OMQOL = Oral Mucositis Quality of Life Measure, MDASI-H&N = MD-Anderson Symptom Inventory—Head and Neck, HNRT-Q = Head and Neck Radiotherapy Questionnaire.

## Supplementary Materials

The following are available online at https://www.mdpi.com/article/10.3390/jcm10184162/s1, Table S1: Characteristics of the Included Studies; Table S2: Cross-comparison of instruments; Table S3: Quality of studies evaluating the validity of PROMS.

## Appendix A

**Table A1:** Medline Search Strategy. *: truncation.

No.	Term
1	*“Head and Neck Neoplasms”/
2	(“head and neck” adj2 cancer$).tw.
3	Exp *Esophageal Neoplasms/
4	Exp *Facial Neoplasms/
5	Exp *mouth neoplasms/
6	exp *Otorhinolaryngologic Neoplasms/
7	exp *Tracheal Neoplasms/
8	((?esophageal or pharyn* or laryn* or hypophary* or oropharyn* or nasopharyn* or trachael or oral or mouth or tongue or nose or ear) adj2 cancer$).mp
9	Or/1–8
10	exp *”signs and symptoms”/
11	symptom$.ti
12	*”Quality of Life”/
13	Nutrition Disorders/
14	(eating adj1 (difficult? or disorder?)).ti,ab.
15	exp Body Weight/
16	salivary gland diseases/or sialadenitis/or xerostomia/
17	exp Taste Disorders/
18	Mucositis/
19	Deglutition Disorders/
20	“dry mouth”.ti,ab.
21	Stomatitis/
22	exp Tooth Diseases/
23	exp Voice Disorders/
24	Speech Disorders/
25	(voice or speech or taste or sialedenitis or xerostomia or dysphagia or stomatitis or mucositis or deglutition or swallowing).ti,ab.
26	Sleep Disorders/
27	“Sleep Initiation and Maintenance Disorders”/
28	Fatigue/
29	fatigue.ti,ab.
30	Muscle Weakness/
31	Trismus/
32	(trismus or spasm$).ti,ab.
33	exp Nausea/
34	Anxiety/
35	Depression/
36	Anger/
37	Depressive Disorder/
38	(anxiety or anger or depression).ti,ab.
39	Body Image/
40	exp self concept/
41	(“body image” or “self-esteem”).ti,ab.
42	spirituality/
43	“well being”.tw.
44	exp Social Adjustment/
45	exp Interpersonal Relations/
46	Stress, Psychological/
47	((emotional or instrumental or social or Information) adj1 support$).ti,ab.
48	exp Adaptation, Psychological/
49	coping.ti,ab.
50	Or/10-49
51	9 and 50
52	*incidence/
53	*Prevalence/
54	exp Registries/sn [Statistics & Numerical Data]
55	(incidence or prevalence).ti,ab.
56	Survivors/sn [Statistics & Numerical Data]
57	Cross-sectional.tw
58	Cross-sectional studies/
59	(observational adj1 (study or studies)).tw
60	OR/52-59
61	51 and 60
62	Limit 61 to (English language and yr = 2004-Current”)
